# Evaluation of automated streaking patterns in urine culture for clinical workflow optimization

**DOI:** 10.1038/s41598-025-15111-8

**Published:** 2025-08-18

**Authors:** Yasuhide Kawamoto, Norihito Kaku, Norihiko Akamatsu, Fujiko Mitsumoto-Kaseida, Takahiro Takazono, Kosuke Kosai, Hiroo Hasegawa, Koichi Izumikawa, Hiroshi Mukae, Katsunori Yanagihara

**Affiliations:** 1 Department of Laboratory Medicine, Nagasaki University Hospital, Nagasaki, Japan; 2 Department of Laboratory Medicine, Nagasaki University Graduate School of Biomedical Sciences, Nagasaki, Japan; 3 Department of Respiratory Medicine, Nagasaki University Hospital, Nagasaki, Japan; 4 Department of Infectious Diseases, Nagasaki University Graduate School of Biomedical Sciences, Nagasaki, Japan

**Keywords:** Automation, Microbiological techniques, Urine, Colony count, Specimen handling, Urogenital diseases, Diagnosis, Infectious-disease diagnostics

## Abstract

Automated streaking systems improve the standardization of microbiological workflows; however, the optimal streaking pattern for single-colony isolation remains uncertain. Seven preinstalled streaking patterns were systematically evaluated using the Copan WASP system to identify the most effective pattern for urine culture. We assessed seven pre-installed streaking patterns using five standard bacterial strains at varying bacterial loads from 10² to10⁷ CFU/mL. The top three patterns were further tested on clinical urine samples collected over three months. Single-colony isolation efficiencies were compared across different bacterial loads and polymicrobial samples. Among standard strains, Single Streak Type 6 (SST6) exhibited the highest single-colony isolation efficiency, followed by Four Quadrants Streak Type 5 and Five Quadrants Streak Type 1. SST6 consistently performed the best under moderate bacterial loads (10–10 CFU/mL). However, in high bacterial load samples (≥ 10⁷ CFU/mL), Four Quadrants Streak Type 5 outperformed SST6 (*p* = 0.003). Additionally, SST6 failed to isolate single colonies of the five species in the polymicrobial samples. Five Quadrants Streak Type 1 showed the lowest coefficient of variation, suggesting greater consistency across different bacterial loads. This study highlighted the critical role of streaking pattern selection in automated streaking systems. While the performance varied depending on the bacterial load and sample complexity, certain patterns demonstrated superior single-colony isolation and consistency. Optimizing streaking patterns is essential for improving reproducibility, enhancing diagnostic accuracy, and standardizing automated microbiological workflows.

**Supplementary Information:**

The online version contains supplementary material available at 10.1038/s41598-025-15111-8.

## Introduction

The automation of laboratory testing has significantly advanced clinical laboratory medicine by improving speed, accuracy, and standardization. While automation has been widely implemented in clinical chemistry and hematology, its application in clinical microbiology has expanded only recently, particularly in areas such as antimicrobial susceptibility testing, species identification, and genetic analysis^1^. One of the most notable developments in this field is total laboratory automation (TLA), exemplified by systems such as Kiestra TLA and WASPLab, which streamline microbiological workflows by integrating inoculation, incubation, and imaging into a unified process, thereby improving operational efficiency and consistency^1,2^.

Modern automated streaking systems function either as standalone units or as integral components of TLA, with the primary objective of optimizing single-colony isolation and minimizing contamination. However, despite advancements in laboratory automation, the optimal method for single-colony isolation remains unknown. We hypothesized that the configuration of the automated streaking pattern—including the number of zigzag motions and quadrant divisions—would affect the efficiency and reproducibility of single-colony isolation depending on bacterial load and sample complexity. Several studies have evaluated the performance of automated streaking systems, primarily by comparing them with manual methods or assessing the differences between various automation platforms^3–8^. However, few studies have systematically examined the impact of multiple pre-installed streaking patterns within a single automated system. Most previous studies have analyzed manual versus automated streaking or assessed streaking efficiency under different sample conditions, making direct comparisons challenging^6^. To address this gap, this study systematically evaluated seven preinstalled streaking patterns within a single automated streaking system under standardized conditions, providing a direct comparison of their efficiencies for single-colony isolation.

Urine culture samples were selected because they are among the most commonly used tests in clinical microbiology. They are particularly well-suited for automation because of their high-throughput and standardized processing steps. Urinary tract infections are among the most frequently encountered infections in clinical practice and contribute significantly to the global burden of infectious diseases^9^. These infections require efficient and accurate microbiological diagnostics to guide the appropriate treatment. Urine cultures require precise single-colony isolation, which is a critical step for accurate downstream analyses such as species identification and antimicrobial susceptibility testing^1,10^. Moreover, urine culture results are reported semiquantitatively, using the number of single colonies to determine the clinical significance of bacteriuria, distinguishing among contamination, colonization, and infection. This highlights the need for precise and reproducible automated streaking methods.

In this study, we evaluated the performance of different pre-installed streaking patterns within an automated streaking system to determine the optimal conditions for single-colony isolation from urine cultures. By addressing this practical yet underexplored aspect of automation, our findings contribute to the ongoing standardization of microbiological workflows and support greater consistency in clinical diagnostics.

## Results

### Single-colony isolation in standard strains

**Fig. 1:**
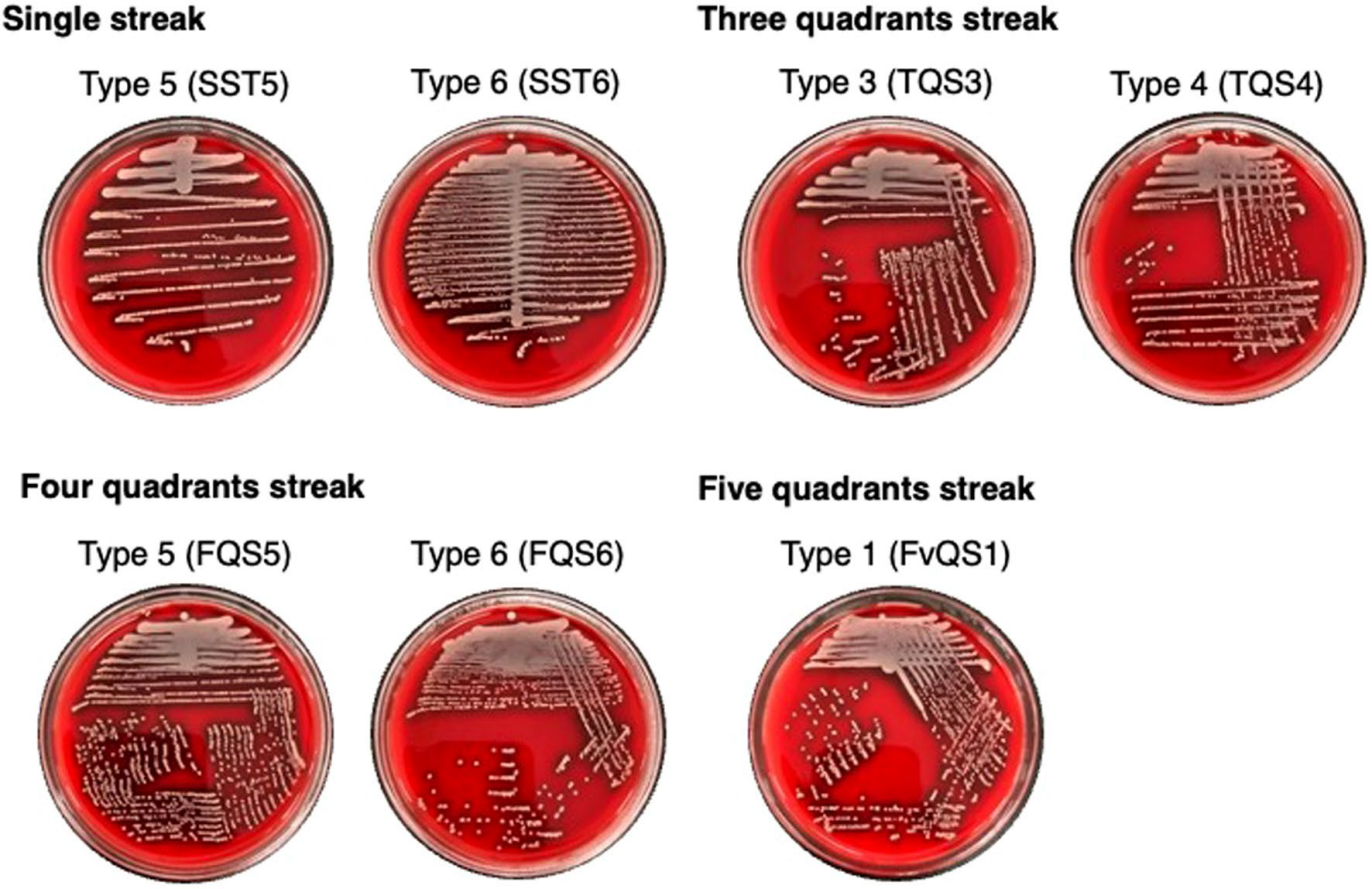
Characteristics of seven pre-installed streak patterns. Representative images of the seven pre-installed streak patterns in the WASP.

**Fig. 2:**
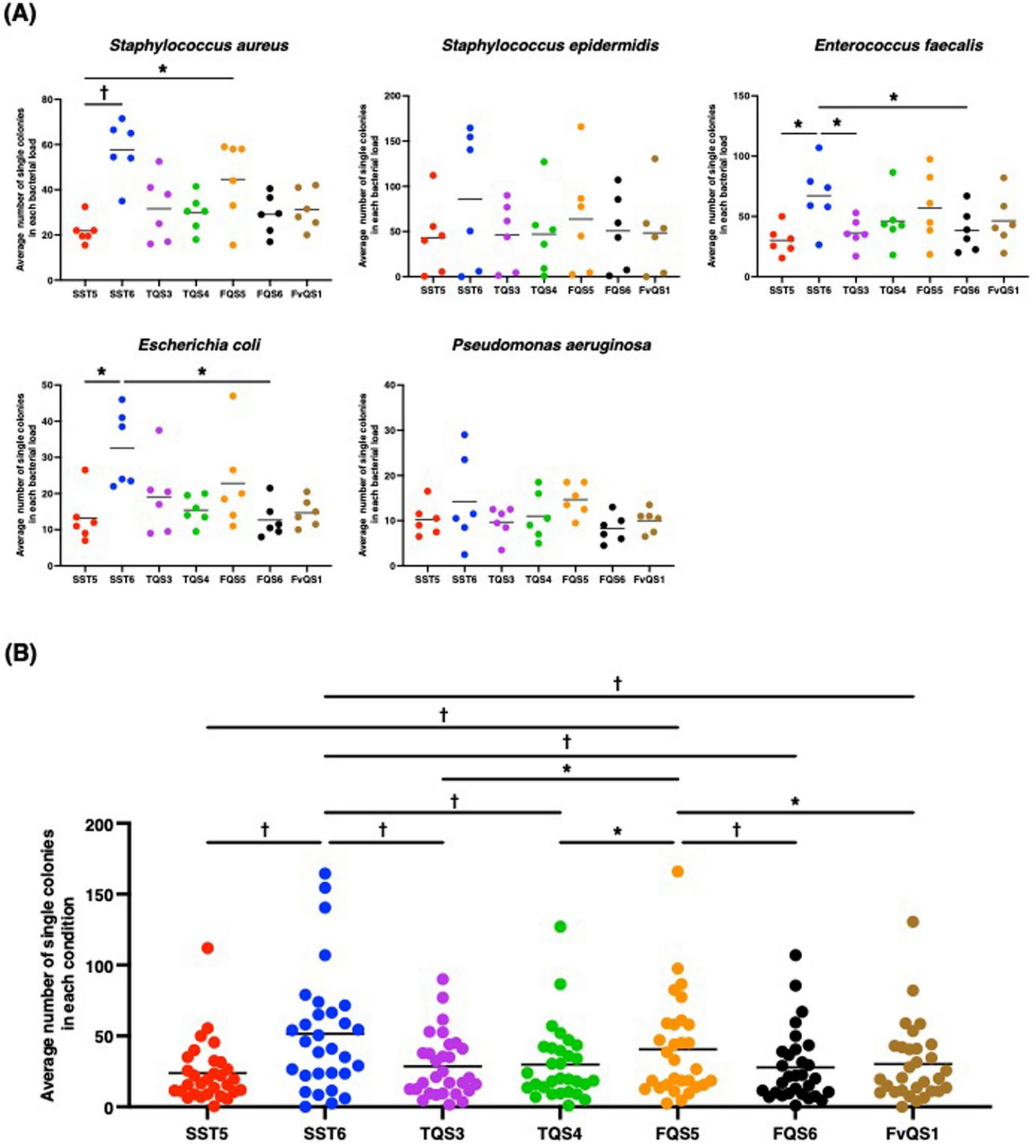
Number of single colonies obtained with standard strains in each bacterial load. The performance of seven streak patterns in the WASP system was evaluated for isolating single colonies using standard strains. The strains included *Staphylococcus aureus* ATCC29213, *Staphylococcus epidermidis* ATCC12228, *Enterococcus faecalis* ATCC29212, *Escherichia coli* ATCC25922, and *Pseudomonas aeruginosa* ATCC27853. The average number of single colonies obtained in each bacterial load in each bacteria (**A**) and all bacteria (**B**).*p < 0.05 and ^†^*p* < 0.001.

Seven pre-installed streak patterns in the WASP system (Beckman Coulter, Inc., CA, USA) (Fig. 1) were evaluated for their ability to isolate single colonies from five standard strains: *S. aureus*, *S. epidermidis*, *E. faecalis*, *E. coli*, and *P. aeruginosa*. Supplementary Fig. S1 shows the number of single colonies obtained at each bacterial load, from 10² to 10⁷ CFU/mL, while Fig. 2 presents aggregated data across all bacterial loads. SST6 achieved the highest single-colony isolation (51.6 $\:\pm\:$ 7.9), followed by FQS5 (40.6 $\:\pm\:$ 6.5) and FvQS1 (30.2 $\:\pm\:$ 5.0) (Fig. 2B). The number of single colonies isolated using SST6 was significantly higher than those isolated using the other streak patterns (Fig. 2B). FQS5 also yielded a higher number of single colonies than all the streak patterns, except for SST6 (Fig. 2B). Accordingly, the overall rankings were SST6, FQS5, FvQS1, TQS4, TQS3, FQS6, and SST5. Based on these rankings, SST6, FQS5, and FvQS1 were selected for further analysis.

### Single-colony isolation in clinical samples

The species detected in the urine cultures are summarized in Supplementary Tables 1 and include *E. coli* (21.8%), *E. faecalis* (19.8%), and *Candida* spp. (12.9%).

**Fig. 3:**
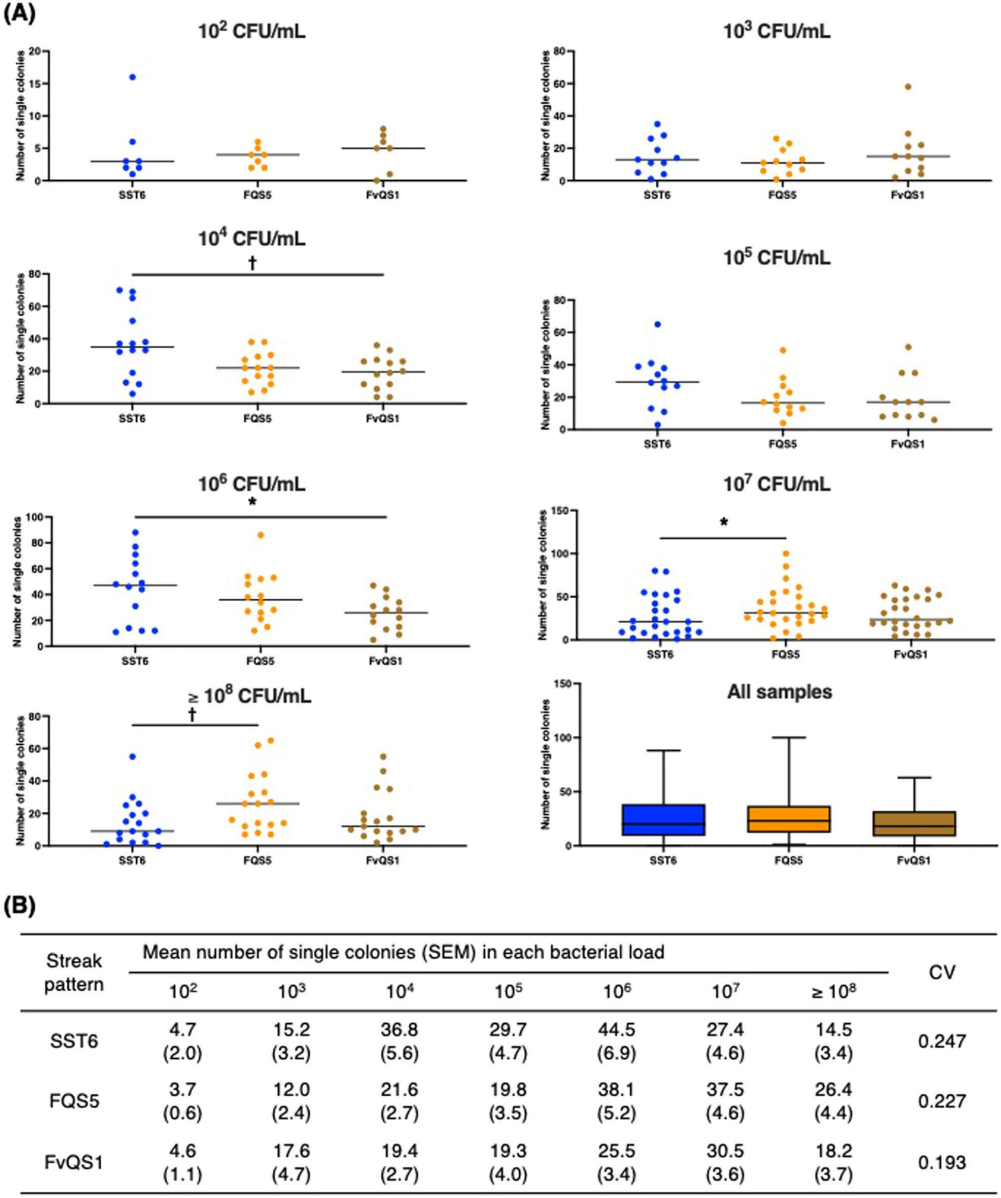
Number of single colonies in the top three streak patterns. The performance of the top three streak patterns in the WASP system was evaluated using urine monomicrobial samples. The number of single colonies was calculated at varying bacterial loads for the samples. In the samples with a bacterial load of 10^4^ and 10^6^ CFU/mL, the mean number of single colonies in SST6 was significantly higher than that in FvQS1 (*p* < 0.001 and *p* = 0.018, respectively) (**A**). In contrast, in the samples with a bacterial load of 10^6^ and ≥ 10^8^ CFU/mL, SST6 showed a significantly lower number of single colonies than FQS5 (*p* = 0.018 and *p* < 0.001, respectively) (**A**). The CV values, in ascending order, were FvQS1 (0.193), FQS5 (0.227), and SST6 (0.247) (**B**). CV, coefficient of variation. *p < 0.05 and ^†^*p* < 0.001.

For clinical samples containing a single bacterial species, the number of single colonies was compared across the top three streak patterns (SST6, FQS5, and FvQS1) (Fig. 3A). In the samples with a bacterial load 10^4^ and 10⁶ CFU/mL, SST6 achieved a significantly higher number of single colonies than FvQS1 (36.8 $\:\pm\:$ 5.6 versus 19.4 $\:\pm\:$ 2.7, *p* < 0.001; 44.5 $\:\pm\:$ 6.9 versus 25.5 $\:\pm\:$ 3.4, *p* = 0.018) (Fig. 3A and B). However, in the samples with a bacterial load of 10^7^ and $\:\ge\:$ 10^8^ CFU/mL, SST6 showed a significantly lower number of single colonies than FQS5 (27.4 $\:\pm\:$ 4.6 versus 37.5 $\:\pm\:$ 4.6, *p* = 0.003; 14.5 $\:\pm\:$ 3.4 versus 26.4 $\:\pm\:$ 4.4, *p* < 0.001) (Fig. 3A,B). Additionally, SST6 failed to isolate a single colony of the five species in the four polymicrobial samples (Table 1).

**Table 1:** The number of single colonies in polymicrobial samples.

Sample	Species	Bacterial load (log_10_[CFU/mL])				Number of single colonies		
		Manual streaking	WASP					
			SST6	FQS5	FvQS1	SST6	FQS5	FvQS1
29	*Morganella morganii*	4.78	4.70	5.18	4.18	4	7	1
	*Enterococcus faecalis*	5.83	5.18	5.18	5.18	30	27	30
55	*Staphylococcus haemolyticus*	4.00	5.18	4.48	5.18	40	8	12
	*Escherichia coli*	3.00	4.70	4.70	5.18	31	7	4
80	*Escherichia coli*	7.65	7.18	8.18	8.18	10	16	21
	*Enterococcus faecium*	7.81	NC	7.18	7.18	0	4	3
120	Coagulase-negative Staphylococci	3.76	2.18	2.18	2.18	4	2	3
	*Streptococcus mitis* group	5.00	4.70	4.18	4.18	33	15	16
152	*Morganella morganii*	7.98	7.18	7.18	7.18	0	18	16
	*Pseudomonas aeruginosa*	12.00	7.18	7.18	7.18	2	4	1
177	*Escherichia coli*	6.60	7.18	7.74	7.74	1	15	16
	*Enterococcus faecalis*	8.31	7.18	7.74	7.74	4	39	36
180	*Pseudomonas aeruginosa*	7.90	7.18	7.74	7.74	2	8	9
	*Corynebacterium striatum*	7.87	7.18	7.18	7.18	0	2	4
187	*Klebsiella pneumoniae*	7.40	6.18	6.18	7.48	12	24	14
	*Enterococcus faecium*	7.08	6.18	5.18	5.18	17	7	8
196	*Enterococcus faecalis*	8.44	7.18	8.18	8.18	0	7	9
	*Escherichia coli*	7.86	7.18	7.18	7.18	0	9	5
203	*Enterococcus faecium*	4.85	4.18	4.18	4.18	14	11	6
	*Escherichia coli*	9.00	4.74	4.18	4.18	24	7	4
	*Enterococcus faecium*	8.00	3.18	3.18	4.74	11	6	3

Not calculable, NC

The coefficient of variation (CV) was calculated to assess the variability in single-colony isolations across different bacterial loads. FvQS1 had the lowest CV (0.193), followed by FQS5 (0.227) and SST6 (0.247) (Fig. 3B).

## Discussion

Single-colony isolation is fundamental for microbiological diagnostics, impacting species identification and antimicrobial susceptibility testing. A previous study has reported that manual streaking methods exhibit variability owing to differences in individual techniques, which can compromise the reproducibility of bacterial isolation^7^. While direct assessments of inter-technician variability in microbiological testing are limited, comparisons between manual and automated streaking systems suggest that automated systems improve reproducibility, enhance single-colony isolation, and increase pathogen detection compared with manual methods, reinforcing the role of automation in standardizing microbiological workflows^7,8,11^ However, few studies have systematically evaluated multiple pre-installed streaking patterns within a single automated system to determine their impact on single-colony isolation^11^.

First, we evaluated the performance of seven preinstalled streak patterns in an automated streaking system, focusing on their ability to achieve single-colony isolation from urine cultures across varying bacterial loads. SST6 consistently yielded the highest number of single colonies among the seven streak patterns tested using standard strains. We selected two single streak patterns (SST5 and SST6) for the standard strains and observed significant differences in their single-colony isolation efficiencies. SST5 yielded significantly fewer single colonies than SST6 for *S. aureus*, *E. faecalis*, and *E. coli* (Fig. 2A) and overall (Fig. 2B). FQS5 ranked second in the single-colony isolation and exhibited a significantly higher number of single colonies than the four-quadrant streak pattern FQS6 (Fig. 2B). A previous study comparing two different streak patterns of the WASP system demonstrated that SST6, which used 27 zigzags, produced significantly more single colonies than Single Streak Type 2, which had only 11 zigzags, highlighting the importance of zigzag frequency in optimizing isolation efficiency^11^. Consistently, our study demonstrated that for both single-streak and four-quadrant streak patterns, a pattern with more zigzag strokes resulted in a higher number of single colonies (SST6 > SST5 and FQS5 > FQS6). These findings and our results suggest that the streaking pattern selection, particularly the number of zigzags, is a key factor influencing single-colony isolation efficiency, reinforcing the need for careful optimization of automated streaking protocols in microbiological workflows.

Although SST6 demonstrated a superior single-colony isolation efficiency when tested against standard strains, its performance varied when applied to clinical urine samples. Among the three streak patterns, SST6 showed the highest number of single colonies at bacterial loads from 10^4^ to 10^6^ CFU/mL). Still, at bacterial loads ≥ 10⁷ CFU/mL, SST6 exhibited a significantly lower single-colony isolation efficiency than FQS5 (Fig. 3A). This suggests that, although SST6 is optimal under moderate bacterial loads, it may not be suitable for cases of heavy bacterial growth, where excessive colony density can interfere with proper isolation. Additionally, SST6 failed to isolate single colonies of the five species in the four polymicrobial urine samples (Table 1). This finding highlights a critical limitation of SST6, namely, its reduced efficiency in separating different bacterial species in the presence of multiple organisms. In contrast, FQS5 demonstrated a significantly better single-colony isolation performance than SST6 at high bacterial loads, suggesting that it may be a more appropriate choice for such conditions. Among the three streak patterns, FvQS1 exhibited the lowest coefficient of variation (Fig. 3B). This suggests that FvQS1 provides the most consistent single-colony isolation across different bacterial loads, making it an ideal choice for standardized clinical workflows.

These findings indicate that although SST6 was the most effective streak pattern when tested with standard strains, the results from clinical samples indicate that FQS5 or FvQS1 may be more suitable choices. Among the evaluated patterns, only SST6 is suitable for semi-quantitative urine culture interpretation, which is essential for clinical diagnosis based on CFU thresholds. In contrast, FQS5 and FvQS1 are better suited for qualitative assessments, such as categorizing growth as rare, some, or numerous, and are more appropriate for samples like pus or wound swabs, where semi-quantification is not routinely performed. The discrepancies between standard strains and clinical isolates highlight the importance of validating streaking patterns in real-world clinical scenarios to ensure optimal single-colony isolation across diverse bacterial loads and sample complexities. Although local practices may vary, our findings provide reproducible evidence that can support laboratories in selecting streaking patterns tailored to sample type and expected bacterial load. Such data-driven selection is particularly valuable in environments adopting total laboratory automation and AI-supported colony recognition.

Several studies have examined the single-colony isolation performance of the Copan WASP system^3–6^. However, most of these studies primarily compared manual streaking and automated methods or different automated systems. Two previous studies have compared two single-streak patterns within the Copan WASP system but did not evaluate three-, four-, or five-quadrant streak patterns, nor did they comprehensively assess multiple preinstalled streaking patterns^5,11^. Another study investigated multiple streaking patterns, but each pattern was applied to different sample conditions, making direct comparisons across streaking patterns difficult^6^. This is the first study to systematically evaluate multiple preinstalled streak patterns within the Copan WASP system using the same set of clinical samples. Unlike manual streaking, in which technician-dependent factors such as inoculum density, streaking pressure, and angle introduce inconsistencies, automated systems minimize variability and improve reproducibility across laboratories^12^. This study provides critical insights into the effect of streaking pattern selection on single-colony isolation efficiency and offers practical guidance for laboratories using the Copan WASP system.Automated streaking systems are integral components of TLA that enhance the standardization and efficiency of microbiological workflows. With advancements in TLA, AI-driven colony recognition and digital plate reading are increasingly integrated into microbiology laboratories, streamlining culture interpretation and result reporting. These systems incorporate image analysis software to automatically interpret colonies on agar, thereby reducing processing time while maintaining diagnostic accuracy^13^. While AI improves colony detection and species identification efficiency, its accuracy relies on well-isolated single colonies. Studies have shown that AI-assisted microbiology benefits significantly from optimized streaking patterns because suboptimal streaking can result in overlapping colonies, thereby reducing the accuracy of AI interpretation^14,15^. This study underscores the critical role of selecting appropriate streaking patterns in automated systems incorporating AI, ensuring optimal single-colony isolation, improved diagnostic precision, and enhanced reproducibility across laboratories.

This study had several limitations. First, this study focused exclusively on urine samples, which may not fully represent the variability in other clinical sample types. Different streaking patterns may yield different results for samples with more complex microbial compositions, such as respiratory samples or wound cultures. The use of only urine samples was a deliberate design choice to ensure consistency in bacterial load, specimen handling, and clinical interpretation. Urine cultures are among the most standardized and high-throughput specimen types in clinical microbiology, making them ideally suited for automated systems. By focusing on urine, we were able to reduce inter-sample variability and ensure a fair comparison of streaking patterns under controlled conditions. Second, this study was conducted using a single Copan WASP system at a single institution, which limits the generalizability of the findings across different laboratory environments. While the results are likely to apply to other laboratories using the same system, inter-laboratory validation studies are needed. Third, this study did not evaluate the effect of different agar media on bacterial colony morphology and growth characteristics. Future studies should explore the effects of cultural media on streaking performance. Fourth, the comparison between manual and automated streaking in this study was limited to a small number of polymicrobial urine samples (Table 1). While manual streaking yielded more single colonies in some cases, these results may reflect the skill of experienced technologists, which is difficult to quantify or reproduce. Although manual methods may outperform automation in specific instances, the WASP system provided consistent outcomes, supporting its utility for reproducibility and laboratory standardization.

In conclusion, this study evaluated seven pre-installed streak patterns in the Copan WASP system and demonstrated that the single-colony isolation efficiency varies based on the bacterial load and sample composition. Specifically, SST6 was the most suitable for semi-quantitative interpretation of urine cultures, essential for clinical diagnosis based on CFU thresholds. In contrast, FQS5 and FvQS1 provided superior performance under high bacterial load and polymicrobial conditions and were better suited for qualitative assessments. These findings emphasize the need to select appropriate streaking patterns to enhance the reproducibility of automated microbiology.

Furthermore, this is the first systematic comparison of multiple pre-installed streaking patterns using the same standard and clinical samples within a single WASP system. The results reveal that performance varies significantly depending on bacterial load and sample complexity, underscoring the practical importance of evidence-based pattern selection. While this study does not introduce mechanistic novelty, it provides essential data to support standardization efforts in laboratories implementing TLA and AI-assisted plate reading systems.

## Methods

### Screening of suitable streak pattern for single-colony isolation using standard strains

A panel of microbiology laboratory technologists reviewed the pre-installed streak patterns of the WASP system (Beckman Coulter, Inc., Brea, CA, USA) and selected seven patterns expected to yield single colonies: Single Streak Type 5 and 6 (SST5 and SST6, respectively), Three Quadrants Streak Type 3 and 4 (TQS3 and TQS4, respectively), Four Quadrants Streak Type 5 and 6 (FQS5 and FQS6, respectively), and Five Quadrants Streak Type 1 (FvQS1). The panel selected the patterns based on their expected performance for single-colony isolation and practical applicability in clinical microbiology settings. All patterns evaluated in this study are included in the standard WASP system and do not require additional licensing. The performance of the seven pre-installed streak patterns for single-colony isolation was evaluated using the following standard strains: *Staphylococcus aureus* (ATCC29213), *Staphylococcus epidermidis* (ATCC12228), *Enterococcus faecalis* (ATCC29212), *Escherichia coli* (ATCC25922), and *Pseudomonas aeruginosa* (ATCC27853). Bacteria stored at − 80 °C were cultured on tryptone soya agar with 5% sheep blood (BD Japan, Tokyo, Japan) at 37 °C with 5% CO_2_ in fully humidified air. After an overnight incubation, the bacteria were suspended in normal saline and diluted to a bacterial load of 1.5 × 10^8^ CFU/mL. Then, we diluted the samples with normal saline to a bacterial load of 1 × 10^2^, 10^3^, 10^4^, 10^5^, 10^6^, and 10^7^ CFU/mL, and all samples were applied to tryptone soy agar with 5% sheep blood (BD Japan, Tokyo, Japan) using the WASP system. A 10-µL loop was employed in the WASP system, as it is the standard loop size designed to ensure consistent sample streaking and colony isolation. After overnight incubation, single colonies on the agar plates were counted. The experiment was tested in duplicate, and the average number of single colonies was determined. Streak patterns were ranked based on the number of single colonies. We selected the top three streak patterns for the clinical study based on their average ranks.

### Evaluation of three streak patterns in clinical samples

**Fig. 4:**
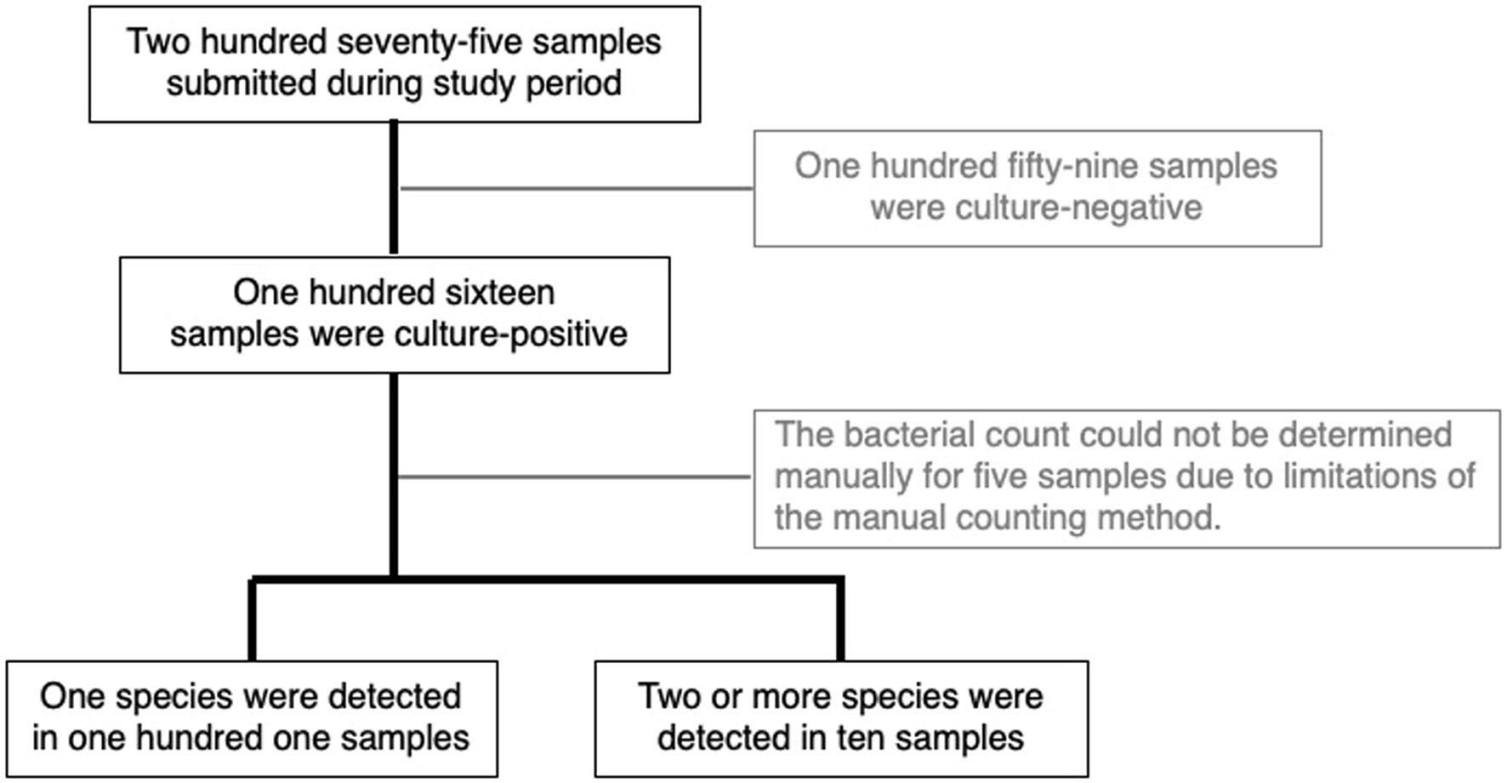
Clinical sample selection for comparison of the top three streak patterns. The performance of the top three streak patterns in the WASP system was evaluated using urine culture samples. Flowchart illustrating the clinical sample selection process.

We evaluated the performance of the top three streak patterns in urine culture samples obtained at Nagasaki University Hospital over three months. Two hundred seventy-five urine culture samples were submitted, and 116 (42.2%) were culture-positive. After excluding the inadequate samples, we evaluated the WASP system using 101 monomicrobial and 10 polymicrobial samples (Fig. 4). The samples were applied to tryptone soy agar by hand, using three streak patterns in the WASP system. After an overnight incubation, the number of single colonies with three streak patterns in the WASP system was evaluated. Additionally, the number of bacteria in each sample was calculated, and the species of bacteria detected in each sample were decided based on the hand method.

### Statistical analysis

All statistical analyses were performed using the GraphPad Prism 10 version 10.4.1 (GraphPad Software LLC, San Diego, CA, USA). The Friedman test was applied to compare the single-colony isolation efficiency across the seven streaking patterns, as the data were nonparametric and involved repeated measurements from the same sample set. Dunn’s post hoc test was performed for pairwise comparisons to identify statistically significant differences between specific streaking patterns. Statistical significance was set at *p* < 0.05. Data are presented as mean ± standard error of the mean.

## Supplementary Information

Below is the link to the electronic supplementary material.

Supplementary Material 1

Supplementary Material 2

## Data Availability

The datasets generated and analyzed during the current study, including colony count data, bacterial load levels, and experimental conditions, are available within the article and Supplementary Information files. Further supporting data are available from the corresponding author upon reasonable request.
