# Supplementary figures and images for: Evaluation of automated streaking patterns in urine culture for clinical workflow optimization
Source: Sci Rep. 2025 Aug 18;15:30175. doi: 10.1038/s41598-025-15111-8 (PMC12361425; doi:10.1038/s41598-025-15111-8)

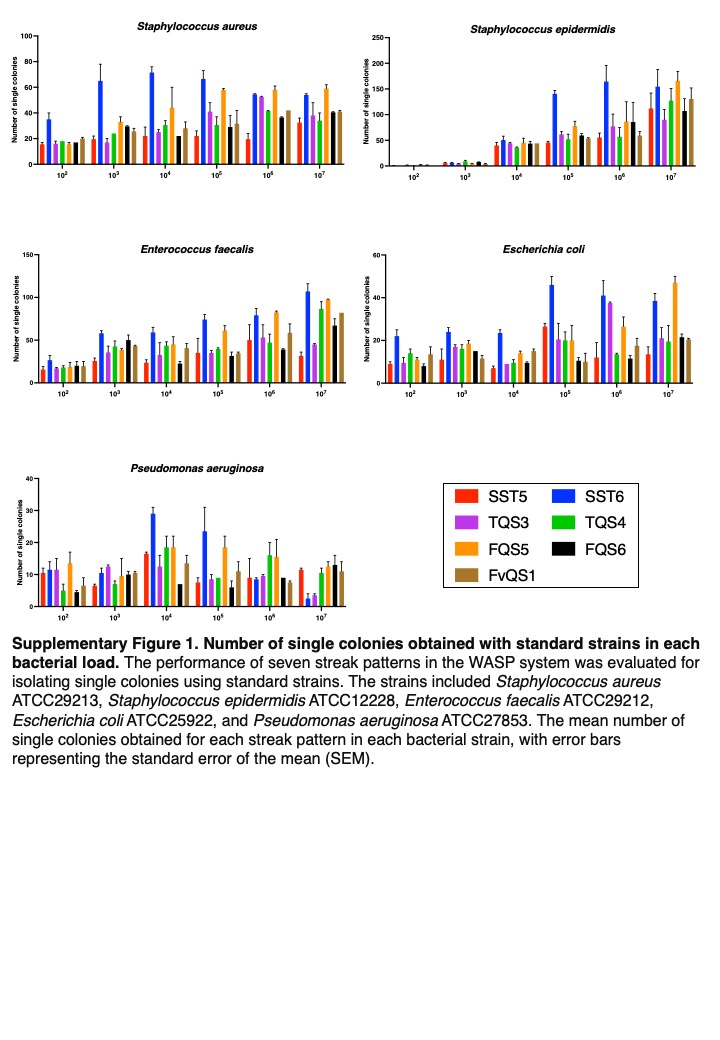

Supplement: Supplementary file 1 — Supplementary Material 1 [file 41598_2025_15111_MOESM1_ESM.jpeg]
